# Sensitive Dentine

**Published:** 1872-06

**Authors:** N. E. Hollace

**Affiliations:** Boston, Mass.


AMERICAN JOURNAL

OF

DENTAL SCIENCE.

Vol. VI. THIRD SERIES--JUNE, 1872. No. 2.

### ARTICLE I.

### *Sensitive Dentine.*

BY N. E. HOLLACE, M. D., BOSTON, MASS.

Before proceeding with this subject, I will mention some of the physiological properties of dentine, and also, briefly refer to the formation of this substance.

This is a subject upon which microscopists and close observers have spent much time, in determining whether there is such a thing as dental tubuli; but as yet, there is a great difference of opinion. Dentine is a very hard dense substance, constituting the inner and larger portion of the crown, and nearly the whole of the root of the tooth. It consists of earthy salts and animal matter, and is harder than bone or cementum; but not so dense as enamel.

It is deposited in concentric layers arranged one within the other, parallel to the surface of the tooth; the last internal layer forming the boundary of the pulp cavity. But in addition to this peculiar structural arrangement, it is, ac cording to the microscopic observations of Prof. R. King Brown and others, composed of minute tubes, or hollow fibers; radiating from the pulp cavity to the periphery of the tooth, giving off in their course numerous branches, sometimes terminating in small cells or corpuscles, and an amorphous or structureless intertubular substance. The tubes radiate from the pulp cavity to the outer surface of dentine, each tube making the principal or primary curve in its course; and presenting when examined with a high magnifying power, numerous secondary undulations, which are less perceptible at the external extremity of the tubes than the middle, and still less in the temporary than in the permanent teeth. These tubes are not mere excavations, but have special parietes; the undulations in them, are ascribed to certain periodic movements in the pulp during the formation of the successive layers of dentine. They are represented as piercing every part of the surface of the pulp cavity. The dental fibres of the crown, in the tooth of the human subject, give off but few branches until they arrive nearly to the outer surface of the dentine; the ramifications become more and more numerous towards the extremity of the root. In the crown they sometimes pass a short distance into the enamel, or terminate in small cavities near the surface, and it is here, or immediately upon the peripheral surface that dentine is most sensitive.

Now, if vessels have been detected in dentine in so many instances, it seems more than probable that they are always present, though too small and attenuated to convey any thing but the thinnest and most serous part of the blood. The delicate sensibility of dentine, especially when in a pathological condition, seems also to favor the opinion that nerve filaments are sent from the pulp to every part of the tissue, traversing no doubt the tubuli, which extend from the central chamber to the periphery. I believe dentine to be supplied with filaments, having found them several times in removing diseased dentine, preparatory to filling, especially from the side of the tooth and, found that the patient experienced much less pain when I applied the excavator to the part nearest the root and cut towards the coronal extremity, than when it was removed in the opposite direction.

The sensibility of dentine is due, no doubt, to the presence of the dentinal fibrils, which, being branches of the dental pulp ramifying the dentinal tubules, terminate at the enamel, in what is termed the sub-enamel membrane; accordingly we find that it is just here that the sensitiveness is most acute in a majority of cases. The pain from sensitive dentine is often exquisite and unbearable, the patient shrinking from the slightest touch of the excavator.

Difficulty often arises in diagnosing a case of sensitive dentine, from one of exposure or almost exposure of the dental pulp. In these cases a knowledge of the location of the pulp cavity, a consideration of the location, size and direction of the cavity of decay, together with the nature of the pain, must be the guide of the practitioner.

The cavity of decay in a tooth may be much deeper in one part of it than in another, and not penetrate to the vicinity of the pulp cavity; bearing in mind that the pulp cavity partakes of the general form of the tooth. If the decay is near the neck of the tooth, a much less depth will suffice to reach the pulp, than when it is situated on a grinding surface. If the cavity of decay is large, or is directed towards the pulp cavity, and deep, the nature of the pain is to be considered. In this there is a marked difference. That proceeding from sensitive dentine is often, as stated above, exquisite and unbearable; while that, from approaching a pulp cavity, is bearable and of a different kind.

There is also another peculiarity in the pain from sensitive dentine; it is that it ceases immediately upon the withdrawal of the instrument, while on the contrary if the pain produced by the excavator in the deeper part of the tooth, remains for a moment or two after the withdrawal of the instrument, it may be ascribed to irritation of the pulp. The usual test is the injection of cold water into the cavity, but it is not reliable, as it will frequently occasion severe pain in cases of sensitive dentine. The causes of sensitive ness of dentine is a contact of the acid secretion of the mouth with the dentinal fibrils.

The partial decomposed material of the teeth, is of necessity infiltrated with these fillings, which are among the principal causes of decay. The remedy for this condition is the local applica tion of an ant-acid in contact with the sensitive dentine. The best of these is chalk, either plain or modified to suit different cases; some patients however, can not bear it; probably carbonas, precip., or levigated chalk.

The precipitated chalk is much finer than the prepared chalk, which is merely levigated; it may be made the basis of an anti-acid.

Tooth powder which should be used from three to five times per day; the philosophy of its operation is as follows: First, the mere brushing of the teeth removes all remains of food and other material, which, remaining, would become putrescent, and increase the acidity. Second, it removes it immediately after eating, thus allowing it no time to produce its deleterious effects. Third, the fineness of the chalk enables it to come in contact with every part of the teeth, and to penetrate into the cavities, thus neutralizing the acid. The object of this treatment is to change the character of the secretions of the mouth from acid to alkaline. This will probably induce a deposition of tartar, which can easily removed, and is preferable to acidity and its concomitants to sensitiveness and decay. In from two weeks to a month it will be generally found that the sensitiveness has disappeared. If the above powder is not strong enough alkaline, the bi-carbonate of soda ʒ, may be added. This is also used as a local application to be placed in the tooth. The brush used should not be too hard or stiff. If this is still not strong enough alkaline, ammonia may be used. If the aq. ammonia is used, care must be taken to prevent it coming in contact with soft tissue, as it is very strong. The vapor also should not be inhaled. An ammoniated mouth wash may be found useful. The condition of the mouth, acid or alkaline, may be ascertained by the use of a little red or blue litimus paper. If this treatment fails resort must be had to an escharolic, which will destroy either the tistue of the tooth itself, or the vitality of the dental fibrils. Creosote will sometimess reduce the sensibility, especially when combined with tannin. It may be applied on a piece of cotton wool or cloth, dipped first into creasote and then into tannin, placing it in the cavity and stopping over with sandraach or gutta percha.

The mere stopping of a cavity with either of those, will often, by the exclusion of the acid portion of the mouth, remove the sensibility in a few days. Comphorated spirits of urine frequently applied in cotton, will reduce the sensibility. Tannin mixed with a solution of gutta percha and chloroform is often useful. Chloroform applied on a small piece of cotton, being a local anæsthetic, will sometimes remove sensibility, if frequently applied.

But the sheet anchor, in difficult cases of sensitive dentine, is the chloride of zinc. This is a powerful escharotic, and acts by decomposing the bone of the tooth to a certain depth all around the cavity, not so deep as the nitrate of silver. It generally induces more or less severe pain, which lasts from one to five minutes, and gradually ceases. If however, instead of ceasing it increases to throbbing, it may be taken for granted that the escharotic has reached the pulp and is irritating it.

It should be withdrawn as soon as this symptom supervenes, when the pain has entirely ceased, the excavation may be performed until all the decomposed tissue is removed, and applied again in the same manner. It is very important in all applications for sensitive dentine, that the mouth, and especially the cavity, be kept as dry as possible. This is the hinging point in the use of chlor. zinc. The mode of application of the chloride, is either on a small piece of cotton dipped into it, or a drop may be taken on the end of a small bent gold wire and introduced into the cavity, just where it is most wanted, that is, immediately under the enamel.

The proper condition for the chlor. use is the deliquesced, which is obtained by merely leaving the stopper out of the bottle and allowing it to absorb moisture from the air, as an exalted condition usually immediately follows its application. It is better that the pain should entirely cease before commencing to excavate.

I think it is Dr. Spalding, of St. Louis, that recommends as his remedy for sensitive dentine, the application of warm burnishers or other instruments, in half worn teeth. Having had no experience in their use I cannot say as to their efficiency. There are other dentists who recommend six inches of good, hard tempered steel, and three inches of Arkansas stone, rubbed into a good edge. It is certain that much depends on the sharpness of the instruments and the manner of excavating. The cutting of a sharp instrument is not so painful as the scratching of a dull one.

The method of operating should be careful, yet prompt and decided, and the strokes should be from the bottom of the cavity, upward and outward, in order to sever the dentinal fibrils in a direction with their course and not contrary to it. Arsenous acid has been and is still used by many for the purpose of reducing the sensibility of dentine, and it is certain that a minute quantity of it introduced into the cavity, will in few hours entirely remove the sensibility. But there is great danger that it will find its way to the dental pulp, and either directly cause its death, or irritate it sufficiently to preclude the possibility of its returning to a healthy condition. For this reason I consider it objectionable, and it should never be used, especially if the sensitiveness proceeds from proximity to the pulp cavity, as in nine cases out of ten (in such cases) it will ultimately cause the death of the pulp, and it is much better to expose the pulp, destroy it, and treat the tooth, than to have the pulp die after the performance of an operation.

The etherial solution of the terchloride of gold acts much in the same manner as zinc chlor., but is open to the objection that both light and air decompose it, precipitating metallic gold, and though the pain it produces is less, yet as it possesses no material advantages over the chlor. zinc, it is not generally used.

The last remedy for sensitive dentine is the administration of a little ether chloroform or choloric ether ; an inhalation of a small quantity ot either of these will prevent the patient from suffering pain, and the inhalation may be repeated if necessary.

